# G-Protein Coupled Receptor Targeting on Myeloid Cells in Atherosclerosis

**DOI:** 10.3389/fphar.2019.00531

**Published:** 2019-05-22

**Authors:** Emiel P. C. van der Vorst, Linsey J. F. Peters, Madeleine Müller, Selin Gencer, Yi Yan, Christian Weber, Yvonne Döring

**Affiliations:** ^1^Institute for Cardiovascular Prevention, Ludwig-Maximilians-University Munich, Munich, Germany; ^2^Department of Pathology, Cardiovascular Research Institute Maastricht, Maastricht University Medical Centre, Maastricht, Netherlands; ^3^Institute for Molecular Cardiovascular Research/Interdisciplinary Center for Clinical Research, RWTH Aachen University, Aachen, Germany; ^4^Munich Heart Alliance, German Centre for Cardiovascular Research, Munich, Germany; ^5^Department of Biochemistry, Cardiovascular Research Institute Maastricht, Maastricht University Medical Centre, Maastricht, Netherlands

**Keywords:** G-protein coupled receptors, myeloid cells, cardiovascular disease, atherosclerosis, therapy

## Abstract

Atherosclerosis, the underlying cause of the majority of cardiovascular diseases (CVDs), is a lipid-driven, inflammatory disease of the large arteries. *Gold standard* therapy with statins and the more recently developed *proprotein convertase subtilisin/kexin type 9* (PCSK9) inhibitors have improved health conditions among CVD patients by lowering *low density lipoprotein* (LDL) cholesterol. Nevertheless, a substantial part of these patients is still suffering and it seems that ‘just’ lipid lowering is insufficient. The results of the *Canakinumab Anti-inflammatory Thrombosis Outcome Study* (CANTOS) have now proven that inflammation is a key driver of atherosclerosis and that targeting inflammation improves CVD outcomes. Therefore, the identification of novel drug targets and development of novel therapeutics that block atherosclerosis-specific inflammatory pathways have to be promoted. The inflammatory processes in atherosclerosis are facilitated by a network of immune cells and their subsequent responses. Cell networking is orchestrated by various (inflammatory) mediators which interact, bind and induce signaling. Over the last years, G-protein coupled receptors (GPCRs) emerged as important players in recognizing these mediators, because of their diverse functions in steady state but also and specifically during chronic inflammatory processes – such as atherosclerosis. In this review, we will therefore highlight a selection of these receptors or receptor sub-families mainly expressed on myeloid cells and their role in atherosclerosis. More specifically, we will focus on chemokine receptors, both classical and atypical, formyl-peptide receptors, the chemerin receptor 23 and the calcium-sensing receptor. When information is available, we will also describe the consequences of their targeting which may hold promising options for future treatment of CVD.

## Introduction

### General Pathology of Cardiovascular Diseases

**FIGURE 1:**
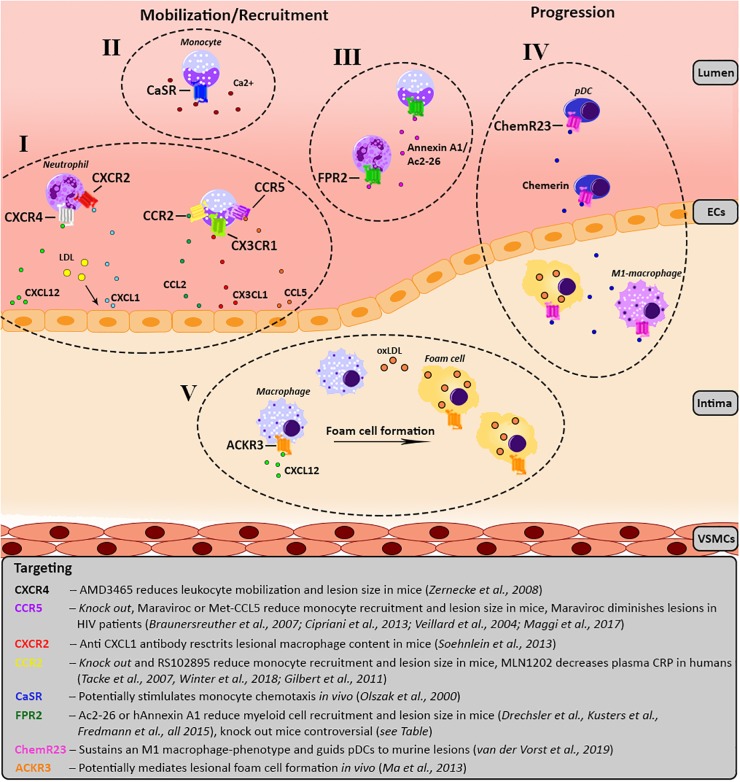
Schematic overview of the involvement of the various GPCRs in atherosclerosis development. For each receptor the key processes, as well as agonists/antagonists are summarized and depicted over three main phases of atherosclerosis development; mobilization, leukocyte recruitment and plaque progression. Receptors of the same GPCR-subfamily are clustered together and categorized from I till V. **(I)** Chemokine receptor CXCR4 causes migration of leukocytes toward its ligand CXCL12. Additionally, upon LDL stimulation CXCL1 is released by endothelial cells causing myeloid cells, which carry CXCR2 on their surface to migrate toward the endothelium. CCL2 and CX3CL1 mediate the recruitment of monocytes expressing CCR2 and CX3CR1, respectively. In line with this, monocytes expressing CCR5 are recruited to the lesion by CCL5. **(II)** Monocytes show a CaSR-dependent increase of chemotaxis toward CCL2 upon stimulation with calcium. **(III)** FPR2 is mostly expressed on myeloid cells and has several contradictory effects, please see Table 1. FPR2-agonists like Ac2-26, an Annexin A1 peptide, and Annexin A1 reduce monocyte/neutrophil recruitment. **(IV)** ChemR23 maintains a M1 macrophage phenotype and stimulates pDC migration and infiltration into atherosclerotic plaques. **(V)** ACKR3 expression is upregulated in lesional macrophages which engulf modified lipids resulting in foam cell formation. ACKR3, atypical chemokine receptor 3; CAD, coronary artery disease; CaSR, calcium-sensing receptor; CCR, C-C chemokine receptor; CCL, C-C chemokine ligand; ChemR23, chemerin receptor 23; CRP, C-reactive protein; CXCR, C-X-C chemokine receptor; CX3CR1, CX3C chemokine receptor 1; CXCL, C-X-C chemokine ligand; CX3CL1, CX3C chemokine ligand 1; FPR2, formyl-peptide receptor 2; HIV, human immunodeficiency virus; LDL, low-density lipoprotein; oxLDL, oxidized LDL; pDC, plasmacytoid dendritic cell.

Cardiovascular diseases (CVDs), with myocardial infarction (MI) and stroke as most common clinical manifestation, remain the leading cause of death worldwide (Hansson, 2005), underpinning the importance of further research into and development of novel therapeutic approaches. Atherosclerosis, a lipid-driven chronic inflammatory disease, has been recognized as the main underlying cause of CVD (Ross, 1999; Hansson, 2005; Braunersreuther et al., 2007a). Endothelial damage by hemodynamic shear stress is a main initiator of atherosclerosis formation, resulting in increased endothelial permeability and hence increased susceptibility for lipid infiltration (Hansson et al., 2015). This damage enables the infiltration of various lipids, like low-density lipoprotein (LDL), into the intima where it is subsequently modified into oxidized LDL (oxLDL) (Ross, 1999; Braunersreuther et al., 2007a). Together with the endothelial damage, accumulation of these modified lipids triggers an inflammatory response, resulting in progressive inflammatory cell infiltration into the sub-endothelial layer (Ross, 1999; Braunersreuther et al., 2007a). During this mobilization stage (Figure 1), predominately monocytes will bind to adhesion molecules on the activated endothelium and subsequently infiltrate into the vessel wall by transmigration (Schumski et al., 2018). Infiltrated monocytes subsequently differentiate into macrophages which phagocyte cell debris and oxLDL, resulting in the formation of foam cells (Hansson, 2005; Charo and Ransohoff, 2006). As foam cells also trigger inflammation by releasing cytokines and chemokines, a vicious circle is created resulting in the continued recruitment and mobilization of leukocytes to the vascular wall. This results in the formation of so called fatty streak lesions which will continue to develop and grow over time. During this progression stage, activated lesional macrophages also secrete matrix metalloproteinases that can digest extracellular matrix components, leading to plaque destabilization. In the end, macrophages become apoptotic due to the continued lipid accumulation and contribute to the formation of necrotic cores (Moore and Tabas, 2011). Besides monocytes and macrophages, also neutrophils have been described to play an important role in the development of atherosclerotic lesions. It has been shown that neutrophils can influence almost every step of this pathology, including endothelial dysfunction, monocyte recruitment, foam cell formation and plaque destabilization (Döring et al., 2015). Eventually, plaque growth or the rupture of lesions resulting in atherothrombosis can cause the artery to occlude. This occlusion will cause ischemia in downstream tissues, resulting in cardiovascular events like stroke or MI (Hansson, 2005).

### Classical CVD-Therapies

Cardiovascular disease-therapy is mostly focussing on mitigation of hyperlipidemia (statins) and management of thrombotic factors (aspirin) to prevent further progression of the disease. Statins are inhibitors of the HMG-CoA reductase, thereby reducing the production of cholesterol and the current golden CVD-therapy (Okopien et al., 2016). A recent meta-analysis of several statin clinical trials indeed confirmed that statin use clearly reduces plasma LDL levels (up to 55–60%) and thereby also resulted in significant reductions in cardiovascular risk (Boekholdt et al., 2014). However, as with a lot of therapies there are also off-target side effects due to the use of statins. It has been shown that statin treatment results in a striking 9% increased risk for the development of diabetes (Preiss et al., 2011). This has led to a debate about the use of statins and especially fuelled the development of adequate alternatives. One of these intriguing new players in the field of hyperlipidemia therapy is monoclonal antibodies against proprotein convertase subtilisin/kexin type 9 (PCSK9). In a physiological condition, PCSK9 interacts with the LDL receptor in the liver to stimulate its degradation and additionally prevention its recycling to the cell membrane (Cohen et al., 2005). Inhibiting PCSK9 thus results in an increased surface expression of LDL receptors that are capable of binding and internalizing LDL particles, thereby reducing the plasma LDL levels. The great potential is demonstrated by the fact that PCSK9 inhibition can cause a 60% reduction of LDL, even on top of the LDL lowering due to statin use, without any indications of serious side effects (Robinson et al., 2015; Stone and Lloyd-Jones, 2015; Zhang et al., 2015). The only major drawback of these monoclonal antibodies is the fact that the production is still very costly and therefore wide-scale usage is not yet feasible.

### Novel CVD-Therapies

Besides above described therapies focussing on lipid modulation, immunomodulation has emerged during the last decades as a promising therapeutic option. Accumulating evidence especially supports the beneficial role of interleukin-1β (IL-1β), tumor necrosis factor (TNF) and IL-6 inhibition (Ridker and Luscher, 2014). All of these cytokines are part of a common pathway. IL-1β is initially produced as an inactive precursor and therefore requires proteolytic cleavage which is mediated by the nucleotide-binding leucine-rich repeat-containing pyrin receptor 3 (NLRP3) inflammasome (Strowig et al., 2012). Inhibition of IL-1β using the monoclonal antibody canakinumab results in the significant reduction of plasma IL-6 and high-sensitivity C-reactive protein (hsCRP) levels, without lowering LDL cholesterol (Ridker et al., 2012). The effect of IL-1β targeting on cardiovascular risk has recently been evaluated in the Canakinumab Anti-inflammatory Thrombosis Outcome Study (CANTOS) trial. This randomized, double-blind, placebo-controlled trial involving stable patients with previous MI showed that canakinumab was effective in reducing plasma hsCRP levels and preventing adverse cardiac events (Ridker et al., 2017). Although this study shows great promise of immunomodulatory therapies, the use of canakinumab was associated with an increased risk of fatal infection or sepsis, despite the exclusion of patients with chronic or recurrent infection. Therefore, more elaborate studies are needed to elucidate the mechanism behind these adverse side effects in order to develop a more specific targeting approach.

### GPCRs as Novel Therapeutic Targets

Although several novel therapies have been explored over the last years, atherosclerosis still cannot be fully reversed by medical treatment, warranting the necessity of innovative therapeutic approaches. Recently, G-protein coupled receptors (GPCRs) have emerged as promising pharmacological targets because of their diverse functions. This is also highlighted by the fact that several recent reviews discussed the targeting of GPCRs in atherosclerosis in a rather general setting (Desimine et al., 2018; Pirault and Back, 2018; Tang et al., 2018; Gencer et al., 2019; Noels et al., 2019). Therefore, in this review we will restrict ourselves to the discussion of the role of GPCRs on myeloid cells in atherosclerosis and CVD and their potential targeting (please also refer to Figure 1).

## G Protein Coupled Receptors (GPCRs)

### General Overview and Classification

G-protein coupled receptors, also known as seven-transmembrane domain receptors or heptahelical receptors constitute, with at least 800 members, the largest family of cell surface receptors (Gloriam et al., 2007; Trzaskowski et al., 2012). These various names can be explained by the fact that these receptors all pass the cell membrane seven times and couple to G proteins to activate internal signal transduction upon activation. GPCRs can bind a wide variety of endogenous ligands, including neuropeptides, amino acids, ions, hormones, chemokines, lipid-derived mediators and ions (Marinissen and Gutkind, 2001; Lagerstrom and Schioth, 2008; Hazell et al., 2012). However, the exact ligands of several receptors remain to be identified, making them orphan GPCRs (Chung et al., 2008).

Classically, the GPCR family was divided into three main classes (A, B, and C) with no detectable sequence homology between these classes (Bjarnadottir et al., 2006). Over the years several subgroups emerged to create a more detailed classification, for example class A which accounts for almost 85% of all GPCRs has been further subdivided into 19 subgroups (A1–A19) (Joost and Methner, 2002). Additionally, the main classification also increased in diversity, creating six main classes based on sequence homology and functional similarity (A-F system) (Foord et al., 2005). Also alternative classification systems have been created, for example the GRAFS system that subdivides the receptors based on phylogenetic analysis into five groups called Glutamate, Rhodopsin, Adhesion, Frizzled/Taste2, and Secretin (Fredriksson et al., 2003).

### Key Pharmacological Concepts

G-protein coupled receptors have distinct binding sites, whereas the main binding site is called the orthosteric binding site, several distinct sites are also susceptible to ligand-binding and are called allosteric binding sites. Depending on the binding site that is used, the ligands can be given the corresponding term orthosteric or allosteric ligand. Binding of allosteric ligands to the receptor will induce a conformational change that influences the affinity or binding potential of orthosteric ligands in a positive (positive allosteric modulator) or a negative (negative allosteric modulator) manner. Before going into detail by discussing the specific GPCRs or sub-families and their respective ligands, it is important for the comprehension of the reader to have a proper definition of several pharmacological concepts (IUPHAR/BPS; Wacker et al., 2017).

An agonist is a ligand or drug that binds to a receptor and alters the receptor state resulting in a biological response. Conventional agonists increase the receptor activity either to the maximum extent (full agonist) or to less than 100% of the maximal response (partial agonist). In contrast, an inverse agonist reduces the receptor activity. For this, the receptor must elicit intrinsic or basal activity already in the absence of the ligand, as the inverse agonist can only then decrease the activity below this basal level. Antagonists do not produce a biological response upon binding to a receptor, but reduces the action of another drug, generally an agonist. Also here, there are two different subtypes being competitive or non-competitive antagonists. Competitive antagonists bind to the same site as the agonist (usually the orthosteric site) on the receptor without causing activation, but thereby blocking the binding of the agonist. This kind of antagonism is reversible by increasing the concentration of agonist to outcompete the antagonist. However, non-competitive antagonists do not compete directly with the binding of the agonist as they bind to an allosteric site on the receptor, resulting in an irreversible effect.

### GPCR Signaling

In the inactive state, GPCRs are bound to a guanosine diphosphate (GDP) associated heterotrimeric G protein complex (G_αβγ_) (Rosenbaum et al., 2009). Upon activation, the GPCR will undergo a conformational change which induces cytoplasmic signal transduction by influencing the G_α_ subunit via protein domain dynamics (Hilger et al., 2018). The activated G_α_ subunit subsequently exchanges guanosine triphosphate (GTP) in place of GDP, triggering the dissociation of the G_α_ subunit from the G_βγ_ dimeric subunit and from the receptor. These two dissociated subunits can then interact with other intracellular effector proteins to further activate various signaling cascades (Digby et al., 2006). G_α_ subunits especially target effectors like adenylyl cyclases, cGMP phosphodiesterase, phospholipase C (PLC), and RhoGEFs (Kristiansen, 2004; Milligan and Kostenis, 2006), while G_βγ_ recruit kinases to the membrane and regulate potassium channels, voltage-dependent Ca^2+^ channels, adenylyl cyclases, PLC, phosphoinositide 3 kinase and mitogen-activated protein kinases (Smrcka, 2008; Khan et al., 2013). As G_α_ has intrinsic GTPase activity, the cellular response is terminated once this subunit hydrolyses GTP again to GDP resulting in the reassociation with G_βγ_. The induced signaling and thus functional consequences of GPCR activation are highly variable and largely depend on the nature and binding efficacy of the ligand (Maudsley et al., 2005; Woehler and Ponimaskin, 2009; Kenakin and Miller, 2010; Zheng et al., 2010; Ambrosio et al., 2011). Currently, there are at least 20 different G_α_ subunits identified, which based on structural and functional similarities can be divided into four families, i.e., G_i_, G_s_, G_q_, and G_12/13_ (Simon et al., 1991). Members of the G_i_ family (e.g., G_αi_, G_αt_, G_αz_) mediate primarily the inhibition of adenylyl cyclase or the activation of phosphodiesterase 6, while members of the Gs family (e.g., G_αs_, G_αalf_) facilitate the activation of adenylyl cyclase. Furthermore, the Gq family members (e.g., G_αq_, G_α11_, G_α14_) are known to activate the kinase PLC, while the G_12/13_ family members (G_α12_ and G_α13_) activate the Rho family of GTPases. Additionally, also G protein-independent interactions have been demonstrated for GPCRs, mainly with β-arrestins (Beaulieu et al., 2005; Lefkowitz and Shenoy, 2005), resulting in the internalization of the receptor into endosomes followed by degradation or recycling of the receptor (Daaka, 2012). Arrestin coupling can also induce activation of downstream effector proteins like mitogen-activated protein kinases or SRC kinases. Interestingly, some GPCRs are even able to activate both G protein-dependent as well as G protein-independent signaling (Feng et al., 2005).

### Ligand Bias Theory

The classical view is that the binding of an agonist to a particular GPCR elicits its effects through a single mechanism of activation, suggesting a single activated confirmational state of the receptor (Stephenson, 1956; Black and Leff, 1983). Recently, by measuring broader networks of signals stimulated by agonists, it has become clear that agonists do not only show quantitative differences (e.g., partial or full agonist, fitting in the classical view) but also functional selectivity (e.g., one ligand selectively stimulates one signal whereas another ligand selectively stimulates a second signal via the same receptor) which is not fitting with this classical view (DeWire and Violin, 2011). This gave rise to the concept of ligand bias or also termed biased agonism (Michel and Charlton, 2018), which especially during the past decade received more appreciation and support. The concept of functional selectivity and ligand bias has been comprehensively reviewed elsewhere (Kenakin and Miller, 2010), even in the context of cardiovascular pharmacology (DeWire and Violin, 2011).

### Importance of GPCRs for Therapeutics

Due to the large variety in functional effects mediated by GPCRs, they have been implicated in a multitude of processes that play a crucial role in atherosclerosis development. GPCRs are the most “druggable” receptor class, as a striking 30–35% of all medicines currently on the market target one of these receptors (Hauser et al., 2017; Sriram and Insel, 2018). This is mainly caused by the fact that most GPCRs have small ligands and thus the corresponding binding pockets in these receptors are also small and therefore relatively easy to target. However, especially within the chemokine receptor family difficulties arise when a single receptor can bind multiple ligands. A small molecule blocking the binding site of one of these ligands does not necessary also blocks the binding of all others (Wells et al., 2006). Besides the use of small molecules also different targeting approaches are being used or at least evaluated, like the modification of ligands or antibodies against specific receptors. As Hauser et al. (2017) recently published a very elegant review of trends in GPCR drug discovery further elucidating the various GPCR drugs and agents that are in clinical trials, we will keep the discussion of this rather limited.

### Focus of This Review

In this review, we will highlight a selection of GPCRs or receptor sub-families mainly expressed on myeloid cells and clearly linked to atherosclerosis. The chemokine receptors, both classical and atypical, formyl-peptide receptors (FPRs), chemerin receptor 23 and the calcium-sensing receptor (CaSR) will be described in detail as they have been shown to play an important role in chronic inflammation and atherosclerosis (Figure 1). When information is available, we will also describe the consequences of their (therapeutic) targeting in CVD.

## Chemokine Receptors

Chemokines (small chemotactic cytokines) and their receptors are multifunctional operators of the immune system facilitating many vital steps of an immune response, such as leukocyte activation, migration, differentiation, phagocytosis and adhesion in addition to their homeostatic roles, such as leukocyte homing (Johnston and Butcher, 2002; Kim, 2004). Chemokines are classified according to their conserved cysteine residues and bind to two types of seven transmembrane receptors: conventional (GPCRs) and atypical chemokine receptors (ACKRs). The main difference between the two types of receptors is the structural inability of ACKRs to couple and thus signal through G proteins (Griffith et al., 2014). With approximately 50 different ligands and 20 receptors, the chemokine/chemokine-receptor family comprises a very complex and also highly dynamic system. Based on the crucial role of this system in various processes that are important in atherosclerosis development and CVD, targeting specific chemokine–chemokine receptors dyads are promising approaches for CVD-treatment (Weber and Noels, 2011).

### Classical Chemokine Receptors

#### CXCL1–CXCR2 Axis

As described before, the accumulation of oxLDL in the vessel is one of the initiating steps of atherogenesis. The oxidation of LDL generates lysophosphatidylcholine, which is the main substrate for the enzyme autotaxin. This enzyme subsequently transforms lysophosphatidylcholine into lysophosphatidic acid (LPA). This LDL-derived LPA will induce the release of CXCL1 from endothelial cells (Zhou et al., 2011). Subsequently, CXCL1 interacts with CXCR2 on neutrophils and classical monocytes, thereby stimulating their mobilization into the blood stream and migration to sites of inflammation. In line with this, systemic absence of CXCL1 or hematopoietic CXCR2-deficiency has been shown to be protective against atherosclerosis in mice by reducing the intra-plaque macrophage accumulation (Soehnlein et al., 2013).

#### CCL2–CCR2 Axis

Another chemokine-axis that has been shown to play an important role during these initial phases of lesion development is the CCL2/CCR2-axis, especially by mediating the mobilization of classical, inflammatory monocytes. Accordingly, CCR2-deficient mice show reduced atherosclerotic lesion formation due to an attenuation of monocytosis (Swirski et al., 2007; Tacke et al., 2007; Weber and Noels, 2011). Recently, it has been shown that there is a striking circadian control of endothelial and myeloid cell activities. This circadian control is part of the daily rhythms, which are controlled by key proteins like CLOCK and BMAL1 (Zhang et al., 2014). A recent study by Winter et al. (2018) could show that such rhythmic control is also present in chronic inflammatory processes of large vessels, thereby mediating rhythmic myeloid cell recruitment. The recruitment of neutrophils and monocytes to atherosclerotic lesions oscillates with a peak during the transition from the activity to the resting phase (Winter et al., 2018). They could show that this oscillating recruitment is regulated by the rhythmic release of myeloid cell-derived CCL2, as blockage of this signaling abolished the oscillatory leukocyte adhesion. Interestingly, the adhesion of myeloid cells to the microvasculature is different than the previously discussed macrovascular effects as here the adhesion peak was reached during the early activity phase. This opens up novel opportunities of well-timed pharmacological targeting of CCL2 in order to modulate the effects on atherosclerosis formation, without disturbing the microvascular cell recruitment (Winter et al., 2018).

Interestingly, deletion of both CCL2 and CX3CR1, or CCR2 and CX3CL1 even further decreased atherosclerosis development compared with single deficiencies in the proteins, which could be attributed to a strongly attenuated monocytosis and hence reduced plaque macrophage accumulation (Combadiere et al., 2008; Saederup et al., 2008). Pharmacological targeting could further confirm these results, as administration of a non-agonistic CCL2-competing mutant (PA508) with increased proteoglycan affinity, or siRNA-mediated silencing of CCR2 in mouse models of MI resulted in reduced recruitment of classical monocytes to the infarcted areas (Liehn et al., 2010; Majmudar et al., 2013). Targeting the CCL2–CCR2 axis has already been evaluated in a phase 2 human clinical trial, where blockage of CCR2 with MLN1202, a specific humanized monoclonal antibody that inhibits CCL2 binding, resulted in reduced plasma CRP levels in patients at risk for CVD (Gilbert et al., 2011). Thereby, targeting of this chemokine-axis remains a promising approach for future CVD therapies.

#### CCL5–CCR5 Axis

Besides recruitment, chemokines and their receptors also play an important role in leukocyte arrest on the endothelium by integrin activation. For example, activated platelets release CCL5 which is subsequently immobilized on the surface of inflamed endothelium, triggering leukocyte arrest (von Hundelshausen et al., 2001). This CCL5-mediated myeloid cell recruitment has been shown to be dependent on sialylation of the receptors CCR1 or CCR5, as deficiency of sialyltransferase St3Gal-IV in mice resulted in decreased monocyte and neutrophil recruitment and reduced atherosclerotic lesion size in a CCL5-related manner (Döring et al., 2014). The potential of targeting CCL5 receptors as therapeutic approach was further validated by studies where CCR5 deficiency (Braunersreuther et al., 2007b), inhibition of CCR5 with maraviroc (Cipriani et al., 2013) or general blockage of CCL5 receptors using Met-CCL5 (Veillard et al., 2004) all showed clearly reduced atherosclerotic lesion size and lesional macrophage content in mice. As maraviroc is an FDA-approved HIV-entry inhibitor, it is already used in the clinic, where it could be observed that treatment of HIV-patients with maraviroc seemed to lower atherosclerotic lesion growth (Maggi et al., 2017). Furthermore, it is interesting to note that there is a correlation between plasma CCL5 levels and the progression of atherosclerosis in patients after acute coronary syndrome (Blanchet et al., 2014). All by all, the CCL5–CCR5 chemokine axis seems a promising therapeutic target, especially as an inhibitor is already in clinical use and proven reduce the atherosclerotic risk.

#### Chemokine Heterodimers

With respect to the development of pharmacological targeting of chemokine receptors, it is intriguing to note that chemokines can also form higher-order complexes with themselves (homomers) or with other proteins (heteromers). For example, CCL5 can form a heteromeric complex with CXCL4 and thereby augmenting the CCL5-stimulated arterial monocyte adhesion (von Hundelshausen et al., 2005). This also has clear implications for atherosclerosis development as selective disruption of the CCL5-CXCL4 heteromer by the cyclic peptide MKEY results in reduced plaque formation in mice (Koenen et al., 2009). Administration of MKEY did not interfere with systemic immune responses, like T cell proliferation of clearance of viral infections, clearly highlighting the potential and specificity of this peptide. Additionally, treatment with MKEY has been shown to preserve heart function and decrease the infarct size in a model of myocardial ischemia/reperfusion injury. Moreover, MKEY treatment resulted in a reduced inflammatory reaction in response to injury, demonstrated by the attenuation of monocyte and neutrophil recruitment. Interestingly, there was also a significant reduction of citrullinated histone 3 in the infarcted tissue, showing that MKEY can also prevent NETosis (Vajen et al., 2018). Another example of a heteromer that stimulates leukocyte adhesion is the complex between neutrophil-borne human neutrophil peptide 1 (HNP1) and platelet-derived CCL5 (Alard et al., 2015). Disruption of this complex with the specific peptide SKY resulted in decreased recruitment of classical monocytes in a murine MI model (Alard et al., 2015). The continued elucidation of the precise physiological and especially pathological functions of various chemokine–chemokine interactions (von Hundelshausen et al., 2017) will further identify novel and interesting targets with clinical potential.

#### CXCL12–CXCR4 Axis

Another important chemokine axis in cell homeostasis, mobilization and immunity is the CXCL12–CXCR4 axis (van der Vorst et al., 2015). For example, systemic treatment of atherosclerosis prone mice with the biglycan CXCR4 antagonist AMD3465 resulted in increased atherosclerosis lesion size compared to untreated controls due to increased neutrophil mobilization (Zernecke et al., 2008). Using cell-specific genetic ablation of CXCR4, endothelial CXCR4 has been shown to promote re-endothelialization after vascular injury and prevent neointimal hyperplasia (Noels et al., 2014) and to limit atherosclerosis development by maintaining the endothelial integrity (Döring et al., 2017). This endothelial barrier integrity was mainly promoted by the signaling of CXCL12–CXCR4 to Akt/WNT/β-catenin resulting in enhanced VE-cadherin expression thereby stabilizing the cellular junctions. Additionally, CXCR4 was shown to be crucial in the maintenance of a normal contractile SMC phenotype. In sharp contrast to the clearly atheroprotective role of vascular CXCR4, its ligand CXCL12 seems to be atheroprogressive as endothelial derived CXCL12 promotes lesion development (Döring et al., 2019). Since the current studies only focused on the role of vascular CXCL12 and CXCR4, it remains to be identified whether and which hematopoietic cells play an important role in the modulation of inflammation by CXCL12–CXCR4. In humans, it has already been shown that both CXCL12 and CXCR4 are associated with CVD. For example, regression analysis demonstrated that the C-allele at *rs2322864* in the CXCR4 locus is associated with an increased risk for coronary heart disease (Döring et al., 2017). Additionally, expression of both CXCR4 and CXCL12 was increased in human carotid atherosclerotic lesions compared to healthy vessels (Merckelbach et al., 2018). Genome-wide association studies further confirmed the importance of CXCL12 by showing that a single nucleotide polymorphism at 10q11 near the CXCL12 locus is independently associated with the risk for coronary artery disease (CAD) (Mehta et al., 2011; Döring et al., 2019). Furthermore, the causal role of CXCL12 as mediator of CAD has been confirmed in the ORIGIN and CARDIoGRAM populations by a mendelian randomization study (Sjaarda et al., 2018). All by all, these data clearly support an important role for the CXCL12–CXCR4 chemokine axis in atherosclerosis development and CVD occurrence.

#### Concluding Remarks

Classical chemokine receptors and their corresponding ligands play a key role in the immune system and have been shown to be drivers and regulators of CVD (please refer to Table 1 for a summary of important studies and their key findings and to Table 2 for an overview of ligand types involved). Interference with this system seems like a very promising therapeutic approach, although this should be carefully designed and has to be context-specific to avoid unwanted, but almost unavoidable, side-effects.

**Table 1:** Consequences of GPCRs targeting in cardiovascular disease *in vivo*.

Receptor	Ligand	Species and tissue or model	Pathophysiology	Results	Receptor effect	References
**Classical chemokine receptors**						
CXCR2	CXCL1	Mouse, injection of anti-CXCL1 antibody	Atherogenesis	Reduced lesion size, decreased macrophage content	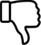	Soehnlein et al., 2013
CCR2	n.d.	Mouse, *Apoe^−/−^Ccr2^−/−^*	Atherosclerosis	Reduced lesion size, decreased monocytosis	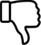	Tacke et al., 2007
	n.d.	Mouse, injection of nanoparticle-encapsulated siRNA targeting Ccr2 in *Apoe^−/−^*	Myocardial infarction model	Attenuated classical monocyte recruitment and infarct inflammation	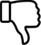	Majmudar et al., 2013
		Mouse, injection of CCR2 inhibitor RS102895 into *Apoe^−/−^*	Atherosclerosis	Reduced myeloid cell recruitment	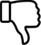	Winter et al., 2018
	CCL2	Mouse, injection of CCL2-competitor PA508 into C57Bl/6	Myocardial infarction model	Attenuated myocardial ischemia/reperfusion injury, reduced classical monocyte recruitment	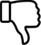	Liehn et al., 2010
	CCL2	Human, specific monoclonal antibody MLN1202 treatment	Atherosclerosis	Decreased plasma C-reactive protein levels	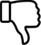	Gilbert et al., 2011
	CX3CL1	Mouse, *Apoe^−/−^Ccr2^−/−^Cx3cl1^−/−^*	Atherosclerosis	Strongly reduced lesion size, decreased monocytosis and plaque macrophage accumulation	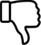	Saederup et al., 2008
CX3CR1	CCL2	Mouse, *Apoe^−/−^Cx3cr1^−/−^Ccl2^−/−^*	Atherosclerosis	Strongly reduced lesion size, decreased monocytosis and plaque macrophage accumulation	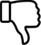	Combadiere et al., 2008
CXCR3	n.d.	Mouse, *Apoe^−/−^Cxcr3^−/−^*	Atherosclerosis	Reduced atherosclerotic lesion size	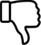	Veillard et al., 2005
	n.d.	Mouse, injection of the CXCR3 antagonist NBI-74330 into *Ldlr^−/−^*	Atherosclerosis	Reduced lesion size, less activated T cells but enrichment of regulatory T cells	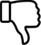	van Wanrooij et al., 2008
	CXCL10	Mouse, *Apoe^−/−^Cxcl10^−/−^*	Atherogenesis and atherosclerosis	Decreased lesion formation, reduced accumulation of CD^4+^ T cells	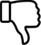	Heller et al., 2006
CCR5	CCL5	Mouse, *Apoe^−/−^St3Gal4^−/−^*	Atherosclerosis	Reduced CCL5-induced myeloid cell recruitment and plaque size	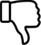	Döring et al., 2014
	n.d.	Mouse, *Apoe^−/−^Ccr5^−/−^*	Atherosclerosis	Reduced lesion size with more stable plaque phenotype	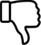	Braunersreuther et al., 2007b
	n.d.	Mouse, injection of CCR5 antagonist Maraviroc into *Apoe^−/−^*	Atherogenesis and atherosclerosis	Decreased atherosclerosis formation by reducing macrophage infiltration	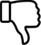	Cipriani et al., 2013
	n.d.	Mouse, injection of CCR5 antagonist Met-RANTES into *Ldlr^−/−^*	Atherosclerosis	Reduced plaque formation, correlated with decreased leukocyte infiltration	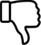	Veillard et al., 2004
	n.d.	Human, CCR5 antagonist Maraviroc treatment of HIV patients	Atherosclerosis	Reduced development of atherosclerosis	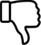	Maggi et al., 2017
	CCL5	Human, plasma	Coronary artery disease	Association between elevated plasma CCL5 levels and the progression of coronary artery disease	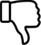	Blanchet et al., 2014
	CCL5–CXCL4 heteromer	Mouse, injection of inhibitory peptide MKEY into *Apoe^−/−^*	Atherosclerosis	Decreased atherosclerosis formation and attenuated monocyte recruitment	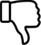	Koenen et al., 2009
	CCL5–CXCL4 heteromer	Mouse, injection of inhibitory peptide MKEY into C57Bl/6	Myocardial infarction model	Decreased infarct size and preserved heart function, attenuated leukocyte recruitment	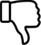	Vajen et al., 2018
	CCL5–HNP1 heteromer	Mouse, injection of inhibitory peptide SKY into C57Bl/6	Myocardial infarction model	Reduced myeloid cell recruitment	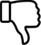	Alard et al., 2015
CXCR4	n.d.	Mouse, injection of CXCR4 antagonist AMD3464 into *Apoe^−/−^*	Atherosclerosis	Increased lesion size due to enhanced neutrophil mobilization	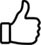	Zernecke et al., 2008
	n.d.	Mouse, *Apoe^−/−^Bmx-Cre^+^Cxcr4^flox/flox^*	Wire-induced injury of carotid artery	Increased neointima formation, due to reduced reendothelialization	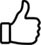	Noels et al., 2014
	n.d.	Mouse, *Apoe^−/−^Bmx-Cre+Cxcr4^flox/flox^*	Atherosclerosis	Increased atherosclerotic lesion formation and disrupted vascular integrity	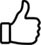	Döring et al., 2017
	CXCL12	Mouse, *Apoe^−/−^Bmx-Cre^+^Cxcl12^flox/flox^*	Atherosclerosis	Reduced plaque size	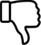	Döring et al., 2019
	n.d.	Human, regression analysis of coronary heart disease cohorts	Coronary heart disease	Associated of the C-allele at rs2322864 with increased risk for coronary heart disease	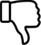	Döring et al., 2017
	CXCL12	Human, carotid atherosclerotic lesions	Atherosclerosis	Increased expression of CXCR4 and CXCL12 in atherosclerotic lesions compared to healthy vessels	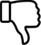	Merckelbach et al., 2018
	CXCL12	Human, genome-wide association studies	Coronary artery disease	Independent association of single nucleotide polymorphism at 10q11 with the risk for coronary artery disease	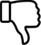	Mehta et al., 2011; Döring et al., 2019
	CXCL12	Human, mendelian randomization study	Coronary artery disease	CXCL12 is a causal mediator of coronary artery disease in humans	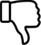	Sjaarda et al., 2018
**Atypical chemokine receptors**						
ACKR1	n.d.	Mouse, *Apoe^−/−^ Ackr1^−/−^*	Atherogenesis and atherosclerosis	Reduced atherogenesis and atherosclerosis formation, with reduced Ccl2 and Cxcl1 expression in aorta	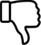	Wan et al., 2015
ACKR3	n.d.	Mouse, *Apoe^−/−^Ackr3^−/−^*	Wire-induced injury of carotid artery	Increased neointima formation and increased lesional macrophage accumulation	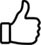	Li et al., 2014
	n.d.	Mouse, *Apoe^−/−^*	Atherosclerosis	ACKR3 expression is upregulated during monocyte-to-macrophage differentiation and thereby enhances phagocytosis	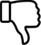	Ma et al., 2013
**Formyl-peptide receptors**						
FPR2	n.d.	Human, coronary lesions	Atherosclerosis	Upregulation of *FPR2* mRNA expression in human lesions compared to healthy vessels	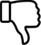	Petri et al., 2015
	n.d.	Mouse, *Ldlr^−/−^ Fpr2^−/−^*	Atherosclerosis	Reduced lesion formation and less macrophage infiltration	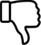	Petri et al., 2015
	n.d.	Mouse, *Fpr2^−/−^* bone marrow into *Ldlr^−/−^*	Atherosclerosis	Decreased atherosclerotic lesion formation and reduced macrophage accumulation	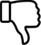	Petri et al., 2015
	n.d.	Mouse, *Apoe^−/−^ Fpr2^−/−^*	Atherosclerosis	Aggravated atherosclerosis formation and increased monocyte recruitment	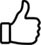	Drechsler et al., 2015
	Annexin A1	Mouse, *Apoe^−/−^ AnxA1^−/−^*	Atherosclerosis	Increased atherosclerosis development and macrophage accumulation	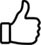	Drechsler et al., 2015; de Jong et al., 2017
	Ac2-26	Mouse, injection of Ac2-26 into *Apoe^−/−^*	Atherosclerosis	Reduced lesion size and lesion macrophage accumulation	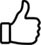	Drechsler et al., 2015
	human Annexin A1	Mouse, injection of Annexin A1 into *Ldlr^−/−^*	Atherogenesis and atherosclerosis	No effect on atherogenesis, but attenuated progression of existing plaques	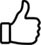	Kusters et al., 2015
	Ac2-26	Mouse, injection of Ac2-26 into *Ldlr^−/−^*	Advanced atherosclerosis	Stabilization of advanced plaques by increasing collagen content while decreasing plaque necrosis	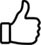	Fredman et al., 2015
	Ac2-26	Mouse, injection of Ac2-26 into *Ldlr^−/−^Fpr2^−/−^*	Advanced atherosclerosis	No beneficial effects of Ac2-26 administration	–	Fredman et al., 2015
	Resolvin D1	Mouse, injection of Resolvin D1 into *Ldlr^−/−^*	Advanced atherosclerosis	Enhanced plaque stability by improved efferocytosis, less necrosis and thicker fibrous cap	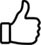	Fredman et al., 2016
	Ac2-26	Mouse, injection of Ac2-26 into C57/Bl6 *and Fpr1^−/−^*	Myocardial infarction model	Reduced acute myocardial injury	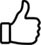	Gavins et al., 2005
**Chemerin receptor 23**						
ChemR23	Chemerin-9 (C9)	Rat, injection of C9 with/without ChemR23 antagonist CCX832 into Sprague-Dawley rats	Hypertension	ChemR23-dependent increased blood pressure	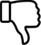	Kennedy et al., 2016
	n.d.	Mouse, *Ob/Ob^−/−^* and *Db/Db^−/−^*	Obesity	Increased serum total chemerin and bioactive chemerin	–	Parlee et al., 2010
	Chemerin-15 (C15)	Mouse, injection of C15 into C57Bl/6	Myocardial infarction model	Reduced heart damage and neutrophil recruitment	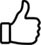	Cash et al., 2013
	Resolvin E1	Mouse, eicosapentaenoic acid supplementation of *Apoe^−/−^* Western Diet of *Apoe^−/−^ ChemR23^−/−^*	Atherosclerosis Atherosclerosis	Reduced atherosclerosis development Increased atherosclerosis development	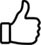	Laguna-Fernandez et al., 2018
	Resolvin E1	Mouse, *ChemR23^−/−^*	Intimal hyperplasia	Increased intimal hyperplasia with more pro-inflammatory macrophages and reduced smooth muscle cell proliferation	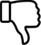	Artiach et al., 2018
	n.d.	Mouse, *Apoe^−/−^ ChemR23^−/−^ Apoe^−/−^ ChemR23^−/−^* bone marrow into *Apoe^−/−^* recipients	Atherogenesis and atherosclerosis	Reduced atherosclerosis development, more M2 macrophages, diminished pDC recruitment Reduced atherosclerosis development	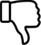	van der Vorst et al., 2019
**Calcium-sensing receptor**						
CaSR	n.d.	Rat, injection of isoproterenol in vitamin D3-induced atherosclerotic Wistar rats	Myocardial infarction model	Increased CaSR expression	–	Guo et al., 2012
	NPSR568	Rat, injection of calcimimetic NPSR568 into spontaneously hypertensive rats	Hypertension	Reduced blood pressure and inhibition of arterial vascular proliferation remodeling	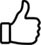	Sun et al., 2018
	Astragaloside IV	Rat, injection of astragaloside IV into Sprague-Dawley rats	Myocardial infarction model	Attenuated myocardial injury and cardiomyocyte apoptosis	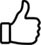	Yin et al., 2019
	Astragaloside IV	Rat, injection of isoproterenol into Sprague-Dawley rat	Myocardial infarction model	CaSR-dependent attenuated cardiac hypertrophy and apoptosis	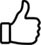	Lu et al., 2018
	Calhex231	Rat, injection of Calhex231 (CaSR inhibitor) into spontaneously hypertensive rats	Hypertension and cardiac hypertrophy	Reduced heart weight to body weight ratio and CaSR levels	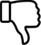	Hong et al., 2017
	Calhex231	Rat, injection of isoproterenol and Calhex231 (CaSR inhibitor) into Wistar rats	Hypertension and cardiac hypertrophy	Amelioration of cardiac hypertrophy and inhibition of autophagy	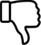	Liu et al., 2016

*n.d., not determined.*

### Atypical Chemokine Receptors

As mentioned before, ACKRs are unable to signal through G proteins but are known to recruit β-arrestin upon ligand binding and are thereby key directors of chemokine driven immune responses as they regulate the bioavailability, internalization, localization as well as the gradient establishment of chemokines (Patel et al., 2009; Ulvmar et al., 2011; Graham et al., 2012; Cancellieri et al., 2013; Bonecchi and Graham, 2016). Moreover, ACKRs can modify the signaling activity of other chemokine receptors via heterodimer formation, thus may also ultimately influence G-protein signaling pathways (Decaillot et al., 2011). Due to their broad-spectrum immunological functions, ACKRs are promising therapeutic targets for the treatment of inflammatory diseases, such as atherosclerosis (Gencer et al., 2019). So far, four types of ACKRs are well recognized: ACKR1 (DARC), ACKR2 (D6), ACKR3 (CXCR7 or RDC-1) and ACKR4 (CCRL1), whereas new members are subject to further investigation: ACKR5 (CCRL2) and ACKR6 (PITPNM3) (Ulvmar et al., 2011). Three members of this family, ACKR1, ACKR2, and ACKR3, are critical for inflammatory responses and will therefore be discussed in greater detail, whereas ACKR4 seems to be primarily involved in homeostatic processes.

#### ACKR1

ACKR1 is expressed on erythrocytes as well as venular endothelial cells and binds plentiful inflammatory chemokines. It is well known that the absence of ACKR1 on erythrocytes causes a Duffy-negative phenotype in African people (Howes et al., 2011; Novitzky-Basso and Rot, 2012; Horuk, 2015). A study by Duchene et al. (2017) showed that Duffy negative individuals exhibited an altered neutrophil phenotype by CCR2, CD16, and CD45 overexpression in comparison to Duffy positive individuals, indicating an amplified defense mode of neutrophils as a result of the lack of ACKR1 on erythrocytes. Considering that ACKR1 binds a wide range of inflammatory chemokines in addition to the characteristic scavenging activity of ACKRs, it is concluded that erythrocyte-specific ACKR1 is a decoy receptor regulating the levels of circulating inflammatory chemokines, such as CCL2 and CXCL8 (Jenkins et al., 2017). Endothelial ACKR1, on the other hand, mediates the internalization of extracellular chemokines and allows their presentation on the cell surface (Novitzky-Basso and Rot, 2012). This process enhances leukocyte recruitment and supports leukocyte-endothelium adhesion, augmenting inflammation. Due to its contrasting roles in different cell types, it is difficult to gauge the impact of systemic ACKR1 deficiency in the context of atherosclerosis. One possibility is that it may lead to a rise in circulating inflammatory myeloid cells, such as monocytes, through an increase in circulating inflammatory chemokines, which would be considered a pro-atherosclerotic event. On the other hand, it may result in a reduction of myeloid cell adhesion to the endothelium, which may in turn decrease lesional macrophage accumulation and thereby limit the development of lesions. Wan et al. (2015) reported an atheroprotective role of ACKR1 deficiency in an apolipoprotein E deficient (*ApoE^−/−^)* mouse model. This was shown to be a result of decreased lesion sizes observed with a decreased inflammatory phenotype in circulating monocytes and macrophages in addition to decreased T-cells in the aortic vessel wall (Wan et al., 2015). This finding highlights a detrimental role of ACKR1 in atherosclerosis. Another study investigating ACKR1 in the context of inflammation through a bone fracture model in mice reported a significant reduction in macrophage numbers around the fractures in ACKR1 deficient mice (Rundle et al., 2013). This outcome was observed with a concomitant decrease in inflammatory markers, such as IL-1β, IL-6 as well as monocyte chemotactic protein-1, confirming a detrimental role for ACKR1 in macrophage recruitment and inflammation. Taken these findings into account, the inhibition of this receptor might be a therapeutic approach in atherosclerosis treatment.

**Table 2:** Types of GPCR-ligands discussed in the review.

Target	Ligand	Type
CCR2	CCL1	Endogenous agonist
	MLN1202	Monoclonal antibody
CCR5	Maraviroc	Antagonist
CXCR2	CXCL1	Endogenous agonist
CXCR4	AMD3465	Antagonist
CX_3_CR1	CX_3_CL1	Endogenous agonist
CCL5–CXCL4	MKEY	Antagonist
CCL5–HNP1	SKY	Antagonist
ACKR3	CXCL11	Endogenous agonist
	CXCL12	Endogenous agonist
	Adrenomedullin	Endogenous agonist
	Bovine adrenal medulla 22	Endogenous agonist
	TC14012	Agonist
FPR2	Annexin A1	Endogenous agonist
	fMLP	Agonist
	Cathepsin G	Endogenous agonist
	Resolvin D1	Endogenous agonist
	Ac2-26	Agonist
	Lipoxin A4	Endogenous agonist
ChemR23	Chemerin (different lengths depending on enzymatic cleavage)	Endogenous agonist/biased agonist (depending on length of ligand)
	Resolvin E1	Endogenous agonist
CaSR	Ca^2+^	Agonist
	Mg^2+^	Positive allosteric modulator
	Cinacalcet	Positive allosteric modulator
	NPS R-467	Positive allosteric modulator
	NPS R-568	Positive allosteric modulator
	NPS 2143	Negative allosteric modulator
	Ronacaleret	Negative allosteric modulator
	Calhex 231	Negative allosteric modulator

#### ACKR2

Similar to ACKR1, ACKR2 also binds numerous inflammatory chemokines. It is expressed on lymphatic endothelial cells, innate-like B cells and some macrophage subsets (Bonecchi and Graham, 2016). Growing evidence discloses an anti-inflammatory profile for ACKR2 with a central role in the resolution of inflammation (Bonavita et al., 2016; Bideak et al., 2018; Massara et al., 2018). ACKR2 is defined as a scavenger receptor for inflammatory chemokines, because ACKR2 deficient mice reproducibly showed increased levels of inflammatory chemokines, like CCL2 (Jamieson et al., 2005; Martinez de la Torre et al., 2005; Whitehead et al., 2007; Collins et al., 2010; Vetrano et al., 2010). The anti-inflammatory properties of ACKR2 are not only limited to its scavenging activities; this receptor is also involved in the regulation of monocyte and macrophage dependent immune responses. For example, ACKR2 deficiency in a murine zymosan A-initiated peritonitis mouse model was shown to promote macrophage efferocytosis, suggesting an important potential function of ACKR2 in atherosclerotic plaques with regards to the efficiency of foam cell efferocytosis (Pashover-Schallinger et al., 2012). Additionally, a *Mycobacterium tuberculosis* disease model lead to rapid death in ACKR2 deficient mice with concomitant increased infiltration of mononuclear cells, e.g., macrophages, into inflamed tissues as well as lymph nodes (Di Liberto et al., 2008). Macrophage infiltration and accumulation in atherosclerotic lesions leads to the progression and eventually growth of plaques. It is therefore of great interest to inhibit these key processes in order to treat atherosclerosis. Considering its roles in macrophage efferocytosis and immune cell infiltration, ACKR2 may be a novel therapeutic target in the research of atherosclerosis treatment. A study conducted by Savino et al. (2012) reported a CCR2-dependent, selective increase in Ly6C^high^ monocyte numbers in circulation as well as secondary lymphoid organs of mice lacking ACKR2 in the non-hematopoietic fragment. This outcome was observed with a delayed graft versus host disease development due to the immunosuppressive activity of the Ly6C^high^ monocytes pointing toward a contrasting role of the receptor in the context of adaptive immune responses. Nevertheless, in the context of atherosclerosis, a rise in inflammatory monocytes in circulation may lead to increased monocyte infiltration and intra-plaque macrophage accumulation, thus result in more advanced lesions. Hence, ACKR2 is a significant immunomodulatory candidate and its roles shall be scrutinized in a cell type and disease model specific manner.

#### ACKR3

ACKR3 is expressed in endothelial cells, marginal B cells, neurons as well as mesenchymal and some hematopoietic cells (Massara et al., 2016). It binds two well-known chemokine ligands, CXCL11 and CXCL12, in addition to adrenomedullin and bovine adrenal medulla 22 (BAM22) (Wang et al., 2018). ACKR3 can signal through β-arrestin and activate extracellular signal-regulated kinase (ERK) as well as phosphoinositide 3-kinase (PI3K)-Akt signaling pathways (Ma et al., 2013). Moreover, ACKR3 can control CXCL12 signaling by either regulating its concentrations or heterodimerization with its alternative receptor CXCR4 (Levoye et al., 2009). Although ACKR3 is crucial in vascular and cardiac development, a number of studies demonstrated its detrimental effects in the context of inflammation.

Research suggests that inflammation caused an increased expression of ACKR3 on immune cells, especially myeloid cells. Infiltrating monocytes in a mouse peritonitis model as well as lesional macrophages in aortic atheroma of mice showed increased ACKR3 expression, pointing toward an inflammatory role of ACKR3 (Ma et al., 2013; Chatterjee et al., 2015). Ma et al. (2013) showed that ACKR3 expression was detected in the macrophage positive area defined by F4/80 positivity within atherosclerotic lesions, whereas this was not observed in the vessel wall of healthy aortas. Moreover, this study showed that whilst undifferentiated THP-1 cells expressed CXCR4 but not ACKR3 mRNA, phorbol 12-myristate 13-acetate (PMA) treatment in THP-1 cells (promoting macrophage differentiation) induced the expression of ACKR3 mRNA whilst downregulating CXCR4 mRNA. Further functional analysis of macrophages with regards to ACKR3 activity was assessed by ACKR3 agonists, such as CXCL12 and TC14012. Treatment of macrophages with these agonists showed increased uptake of FITC-labeled *E. coli,* demonstrating a significant increase in cellular phagocytosis. This effect was abolished by siRNA silencing of ACKR3, confirming that the observed phagocytosis was a result of ACKR3 activity. These findings were endorsed by increased uptake of acetylated LDL by the macrophages stimulated with the same ACKR3 agonists. Another study by Chatterjee et al. (2015) showed that monocytes in the peritoneal fluid of mice with peritonitis showed enhanced CXCR4, ACKR3, and CXCL12 expression, also suggesting that this axis plays an important role in monocyte function during inflammation. Furthermore, ACKR3 was shown to promote monocyte survival and adhesion onto a CXCL12 rich platelet surface as well as the phagocytic activity and foam cell formation of macrophages (Chatterjee et al., 2015). In line with these results, ACKR3 is suggested to support monocyte to macrophage differentiation through CXCL12 activity (Sanchez-Martin et al., 2011). This was supported by the significant reduction of CD136 expression of human monocytes upon both CXCR4 and ACKR3 antagonist treatment. In the same study, monocyte differentiation into CD136^+^ macrophages was shown to be inhibited by means of CXCL12 neutralization as well as CXCR4 and ACKR3 blocking. Moreover, exogenous CXCL12 dependent M-CSF production by the monocytes was partially inhibited by CXCR4 and ACKR3 antagonists, further confirming CXCL12, CXCR4, and ACKR3 dependent regulation of monocyte to macrophage differentiation. Altogether, these findings suggest that ACKR3 promotes atherosclerosis by supporting monocyte and macrophage driven inflammatory processes. Therefore, its inhibition might be a valuable therapeutic target in order to interfere with key events driving atherosclerosis.

#### Concluding Remarks

Without a doubt, ACKRs play crucial roles in the regulation of immune responses and therefore offer significant therapeutic targets in order to control the inflammatory processes. Nevertheless, their wide array of functions establishes a great complexity, making it very difficult to determine individual targets. Thus, it is of great importance to scrutinize and understand the biology of ACKRs CVD (please refer to Table 1 for a summary of important studies and their key findings and to Table 2 for an overview of ligand types involved).

## Formyl-Peptide Receptors

Formyl-peptide receptors (FPRs) belong to the group of pattern recognition receptors (PRRs) and comprise a family of chemoattractant GPCRs involved in host defense against bacterial infections and clearance of cell debris. FPRs are well conserved among mammals (Ye et al., 2009) and are mainly present on myeloid cells such as neutrophils (except FPR3) and monocytes (He and Ye, 2017). In addition to myeloid cells, astrocytes, microglia, hepatocytes, and immature dendritic cells express FPR1, whereas FPR2 is also expressed on epithelial cells, hepatocytes, microvascular endothelial cells, and smooth muscle cells (He and Ye, 2017). FPRs were originally discovered as receptors that bind highly conserved *N*-formyl methionine-containing protein and peptide sequences of bacterial and mitochondrial origin (Forsman et al., 2015). For example, one of the most potent agonists for FPR1 is the *Escherichia coli*-derived peptide *N*-formyl methionyl-leucyl-phenylalanine (fMLF) (Ye et al., 2009), while the most prominent bacterial FPR2 ligands are the *staphylococcal*-derived phenol-soluble modulins (PSMs) (Kretschmer et al., 2015). However, it has become evident that FPR1 and FPR2 recognize a variety of structurally diverse ligands including many host-derived endogenous agonists (see also Table 2) such as Annexin A1, Resolvin D1, Cathepsin G, and the cathelicidin LL37 (*mouse: Cramp*) all of which have been associated with inflammation and/or resolution in mice and man (He and Ye, 2017; Filep et al., 2018). Hence, FPRs may exert ambivalent effects during leukocyte recruitment and in (chronic) inflammatory conditions such as atherosclerosis.

### Role of FPR2 – Annexin A1 in Atherosclerosis Development

Studies on human atherosclerotic plaque specimens supported the notion of the involvement of FPRs in lesion development by pointing at defective resolution within these lesions (Fredman et al., 2016). Additionally, FPR2 mRNA expression was upregulated in human samples from coronary lesions in comparison to healthy vessels (Petri et al., 2015). Similarly, mice deficient for the low-density lipoprotein receptor (LDLR) and FPR2 exhibited decreased atherosclerosis development and less monocyte infiltration and foam cell formation compared with control animals. Analogous results were obtained in *Ldlr^−/−^* mice transplanted with FPR2-deficient bone marrow, here dampened activation of lesional macrophages was also attributed to the lack of FPR2 (Petri et al., 2015). These findings support *in vitro* work from Lee et al. (2013, 2014) showing that oxLDL and serum amyloid-2 mediate foam cell formation via FPR2. Hence, one could argue that agonists, which mediate lesional macrophage activation via FPR2 disturb resolution. However, FPR2 expression on vascular smooth muscle cells (VSMCs) seems to stabilize atherosclerotic lesions suggesting a diverse role of FPR2 on hematopoietic versus vascular cell types. Still, specific agonists or antagonists, which mediate one or the other response, were not investigated in this study (Petri et al., 2015). In contrast, *Apoe^−/−^* mice which also lacked FPR2 or Annexin A1 showed enhanced atherosclerotic lesion development, increased myeloid cell recruitment and adhesion to the inflamed vessel wall. One explanation focusses on the observation that Annexin A1/FPR2 interaction seems to tightly control and inhibit integrin activation (Drechsler et al., 2015; de Jong et al., 2017). In line, treatment of *Apoe^−/−^* or *Ldlr^−/−^* mice with Annexin A1 or the Annexin A1 fragment Ac2-26 reduced atherogenesis by decreasing necrosis, mediating efferocytosis and supporting fibrous cap stability (Drechsler et al., 2015; Kusters et al., 2015). Equivalent results were obtained in *Ldlr^−/−^* mice with advanced atherosclerosis, which were treated with the agonist Ac2-26 packed into nanoparticles that targeted type IV collagen to ensure deposition in atherosclerotic lesions. Plaques of animals treated with Ac2-26 nanoparticles displayed reduced macrophage numbers, smaller necrotic core sizes, and higher amounts of anti-inflammatory interleukin 10 compared to control animals. On the contrary, when treating *Ldlr^−/−^ Fpr2^−/−^* mice with Ac2-26, the protective effects were abolished suggesting an important role of FPR2 on myeloid cells in mediating arterial (lesional) resolution through interaction with Annexin A1 (Fredman et al., 2015).

### Pro-resolving Lipid Mediators

Specialized pro-resolving lipid mediators (SPMs) including the resolvins are derived from the ω-3 PUFAs eicosapentaenoic acid (EPA) or docosahexaenoic acid (DHA). They have important roles in the resolution of inflammation, either via their own GPCRs or by modulating GPCRs for ω-6 PUFA. For example, resolvin E1 (RvE1) enhances the phagocytosis of apoptotic neutrophils via ChemR23 (please also see section “ChemR23 and Resolvin E1 – Mechanisms of Resolution”) and inhibits the infiltration of neutrophils by antagonizing LTB4 or leukotriene B4 receptor 1 (BLT1). Resolvin D1 (RvD1) instead has been shown to bind to two GPCRs, namely, the orphan receptor, GPR32, and the lipoxin receptor, FPR2/ALX through which it mediates its pro-resolving effects (Jannaway et al., 2018). In line with this, if the endogenous agonists RvD1 was administered to *Ldlr^−/−^* mice during the transition phase of atherosclerotic lesions from early into advanced plaques. Fredman et al. (2016) could show that RvD1 enhanced lesional efferocytosis, and decreased plaque necrosis compared with vehicle controls. Similarly, repetitive administration of endogenous agonists Resolvin D2 to *Apoe^−/−^* mice prevented atheroprogression, though most likely mediated via the G-protein coupled receptor 18 (Viola et al., 2016). These findings illustrate the therapeutic potential of pro-resolving FPR agonists to restore defective resolution, which is most likely mediated through myeloid cells in atherosclerotic lesions.

### FPR Signaling and MI

Consistently, a protective role of Annexin A1 and its mimetic peptides could also be demonstrated in experimental models of ischemia-reperfusion injury, e.g., in a mouse model of MI (Gavins et al., 2005). Moreover, Ferraro et al. (2019) for example examined to what extent endogenous control of inflammation resolution and its therapeutic stimulation enables improved cardiac function in the absence and presence of Annexin A1. They showed that myeloid cells infiltrating at early stages post MI deliver Annexin A1 hereby terminating inflammation and promoting healing through macrophages with an angiogenic phenotype with release of VEGF-A. They could further reveal similar protective functions of Annexin A1 in a model of MI in pigs, hence demonstrating that Annexin A1 facilitated cardiac angiogenesis and myocardial repair (Ferraro et al., 2019).

### FPR Signaling Complexity

Other FPR agonists such as Cathepsin G (Ortega-Gomez et al., 2016) and LL37/Cramp (Döring et al., 2012; Wantha et al., 2013) clearly mediate pro-atherogenic effects by enhancing monocyte adhesion and recruitment, though one cannot exclude that these functions may partly be mediated by other receptors. As FPRs recognize both pro-inflammatory and pro-resolving signals, the question remains how one receptor can mediate opposing responses. In this context, Cooray et al. (2013) suggested that anti-inflammatory, but not pro-inflammatory signals activate homodimerization of FPR2, which, in turn trigger the release of anti-inflammatory mediators such as interleukin 10. Heterodimers instead can transduce, e.g., pro-apoptotic signals, explaining why the same receptor system may integrate diverse signals (Cooray et al., 2013). Another plausible option is the concept of biased agonism (please also see section “GPCR Signaling”) (Michel and Charlton, 2018) describing that agonists/antagonists might activate specific receptor domains, thereby promoting downstream responses, which at least in part do not overlap. As an example, the small lipid lipoxin A_4_ has been shown to activate FPR2 by interacting with its extracellular loop III (Chiang et al., 2000), while, e.g., serum amyloid A responses were reliant on extracellular loops I and II (Bena et al., 2012). Hence, all of the latter should be considered in the context of designing potential new therapeutics triggering resolution via FPRs.

### Concluding Remarks

Formyl-peptide receptors have evolved to be a class of receptors that recognize a broad range of structurally distinct ligands and are expressed by a variety of cell types. Many studies have also shown that FPR function is not restricted to host defense against microbes, but also impacts on chronic inflammatory disease such as atherosclerosis and autoimmune diseases or even cancer. Most interestingly, FPR2 does not only mediate pro-inflammatory but also resolution processes and return to homeostasis. While these findings greatly expanded the scope of the pharmacology and biology of FPRs, a better understanding of how FPRs recognize and respond to distinct ligands is needed to explore their further potential as therapeutic targets (please refer to Table 1 for a summary of important studies and their key findings and to Table 2 for an overview of ligand types involved).

## Chemerin Receptor 23

The chemerin receptor 23 (ChemR23; chemokine-like receptor 1, CMKLR1) is a class A (rhodopsin-like) GPCR expressed on the surface of immune cells subtypes such as dendritic cells (Vermi et al., 2005), monocytes and macrophages (Herova et al., 2015). It is therefore expressed in spleen and lymph nodes, but also in the skin, adipose tissue (Goralski et al., 2007; Goralski and Sinal, 2009) and lung (Wittamer et al., 2003; Roh et al., 2007). Functionally, ChemR23 – mostly through its *bona fide* ligand chemerin- mediates immune cell activation and chemoattraction (Carlino et al., 2012; Rourke et al., 2013). The gene encoding for ChemR23 is called *CMKLR1* (non-human = *cmklr1*), first cloned in 1996 by Gantz et al. (1996) and, under the name of ChemR23, in 1998 by Samson et al. (1998). Another gene encoding G-protein coupled receptor 1 (GPR1) was proved to share a common ancestor with *CMKLR1* (Vassilatis et al., 2003) with a sequence identity of 37% (Kennedy and Davenport, 2018). Therefore, it is designated as chemerin receptor 2. The corresponding human sequence for ChemR23 and GPR1 share 80% sequence identity with its corresponding murine genes (Kennedy and Davenport, 2018). ChemR23 has two known ligands in mouse and human, namely chemerin and Resolvin E1 (RvE1). Based on its similarities with GMKLR1, Barnea et al. (2008) were the first to identify chemerin as a ligand for GPR1. GPR1 can act to modify glucose homeostasis during obesity, in line with known functions of chemerin (Rourke et al., 2015). However, as yet it is largely unknown what G protein pathway it activates, only few studies showed chemerin modestly induced calcium release (Barnea et al., 2008) or RhoA signaling (Rourke et al., 2015), more investigation are warranted to unveil the downstream pathways. In this review, we mainly discuss the role of ChemR23 in CVD.

### Chemerin and Its Functions

The adipokine chemerin is encoded by the *RARRES2* (non-human = *rarres2*) gene (Nagpal et al., 1997; Busmann et al., 2004). After being translated into the circulating pro-chemerin, the protein undergoes extensive enzymatic processing. It has been shown that the serine proteases, cathepsin G and elastase, are the main enzymes responsible for the conversion of pro-chemerin into its active form (Wittamer et al., 2005; Ortega-Gomez et al., 2016). The resulting protein variants differ in length and functional properties (Meder et al., 2003; Wittamer et al., 2003). Depending on the chemerin variant binding to ChemR23, the receptor couples to a different subtype of Gα_i_ or isoform of Gα_o_ (Wittamer et al., 2004). Regarding downstream-signaling via ChemR23, it could be shown that the variants C9 (or chemerin-9) and 13 were more potent in inhibiting G protein-dependent cAMP, but less potent in inducing β-arrestin compared with human chemerin 21-157. In summary injection of C9 into rats increased blood pressure via ChemR23 but not via GPR1 mediated signaling and could be inhibited by applying the ChemR23 specific antagonist CCX832 (Kennedy et al., 2016). This lead to the conclusion that shorter C-terminal fragments of chemerin seem to impose a strong bias toward activating G protein coupled signaling. Therefore, signaling via ChemR23 cannot be pinpointed to induce neither purely pro-inflammatory nor anti-inflammatory effects.

### ChemR23–Chemerin Axis in CAD

Studies in animal models of CAD for example showed that expression of both ChemR23 and chemerin were induced in mice which were fed a high fat diet (Roh et al., 2007). Human studies also show elevated plasma concentrations of chemerin and their association with an increased risk of hypertension (Kennedy et al., 2016; Watts et al., 2018), a higher body mass index and blood pressure in patients with type 2 diabetes mellitus compared to healthy controls (Yang et al., 2010). Increased expression levels of ChemR23 were also described in perivascular fat tissue and correlated with increased blood pressure (Neves et al., 2015). In line, augmented expression levels of both ChemR23 and chemerin have been shown in atherosclerotic plaques of human patients and in mouse models of vascular inflammation and a positive correlation between chemerin expression in perivascular adipose tissue and atheroprogression has already been demonstrated (Kostopoulos et al., 2014). Another hypothesis includes an influence of adipokine expression in the heart and vasculature and subsequent plaque progression (Spiroglou et al., 2010). Specifically, it was already found that chemerin expression in human epicardial adipose tissue was positively correlated with the severity of coronary atherosclerosis (Gao et al., 2011). In addition, plasma chemerin levels are associated with markers of inflammation and are significantly higher in CAD patients, which do not receive low dose aspirin treatment. The latter does also reduce pro-inflammatory cytokine secretion by macrophages, which may lead to reduced chemerin secretion by adipocytes and may be a reason for the lower chemerin levels in the circulation of CAD patients on low dose aspirin (Herova et al., 2014).

In contrast, Cash et al. (2013) reveal a protective role of certain chemerin variants in a model of acute MI by preventing excessive neutrophil infiltration. Chemerin-15 induced signaling via ChemR23 was also described to increase efferocytosis in macrophages *in vitro* and in an *in vivo* model of peritoneal inflammation (Cash et al., 2010) and was shown to reduce acute intravascular inflammatory events in murine cutaneous wounds (Cash et al., 2014). In a recently published dietary intervention study, anti-atherosclerotic effects of ChemR23 were outlined using a ChemR23 knock-out mouse model on an *Apoe^−/−^* background (Laguna-Fernandez et al., 2018). A deficiency in ChemR23 (*Apoe^−/−^ ChemR23^−/−^*) seemed to accelerate atherogenic signaling in macrophages, induced cholesterol uptake and phagocytosis and lead to an increased lesion size and reduced plaque stability, hence claiming that a functional receptor mediates atheroprotective signaling (Laguna-Fernandez et al., 2018). Contradictory, in a very recent publication from our group we saw that hematopoietic ChemR23-deficiency increases the proportion of alternatively activated M2 macrophages in atherosclerotic lesions and attenuates pDC homing to lymphatic organs and recruitment to atherosclerotic lesions, which synergistically restricts atherosclerotic plaque formation and progression (van der Vorst et al., 2019). Nevertheless, *ChemR23^−/−^* VSMCs exhibited a significantly lower proliferation rate compared with VSMCs derived from *ChemR23^+/+^* mice while ChemR23-deficient peritoneal macrophages from had significantly higher mRNA levels of pro-inflammatory cytokines compared with *ChemR23^+/+^* macrophages. Finally, conditioned media (CM) transferred from *ChemR23^−/−^* macrophages to VSMCs significantly increased VSMC proliferation compared to treatment with CM from *ChemR23^+/+^* macrophages at least *in vitro*. These results assert dual signaling effects to ChemR23 depending on cell type (VSMCs versus macrophages) expressing the receptor (Artiach et al., 2018), pointing at a diverse role of the receptor on hematopoietic versus vascular cells in atherosclerotic lesion development. An alternative hypothesis suggests that anti-inflammatory effects of chemerin in atherosclerosis are exerted via reduced adhesion to the affected vascular endothelium. One study could show a downregulation of vascular cell adhesion molecule – 1 following chemerin treatment in human umbilical vein endothelial cells and rat aorta and consequently, a reduced monocyte adhesion to the arterial wall (Yamawaki et al., 2012). In conclusion, while chemerin/ChemR23 seems to exert more pro-inflammatory effects on hematopoietic cells, its presence on vascular cells seems to point at an anti-inflammatory role of this ligand receptor pair. However, the relative abundance of pro- versus anti-inflammatory ligands which may also compete with each other and their highly tissue specific expression patterns are expected to further shape these diverse cellular responses in different stages of (chronic) inflammation such as atherosclerosis.

### ChemR23 and Resolvin E1 – Mechanisms of Resolution

RvE1, a metabolite of EPA (a type of polyunsaturated fatty acids), plays an important role in the return to tissue homeostasis (Schwab et al., 2007; Gao et al., 2013; Hasturk et al., 2015) and is suggested to exhibit anti-inflammatory and pro-resolving effects via ChemR23 or leukotriene B4 receptor 1 (BLT1) (Arita et al., 2005b; Arita et al., 2007) (please also see section “Pro-resolving Lipid Mediators”). RvE1-dependent blockage of VSMC migration, a critical process in the progression of atherosclerosis, and switching into a protective anti-atherosclerotic phenotypic in VSMCs, confer an anti-inflammatory role of vascular ChemR23 signaling (Ho et al., 2010). Moreover, RvE1 rescues impaired neutrophil phagocytosis, oxidized LDL uptake and phagocytosis of macrophages, promotes phagocytosis-induced neutrophil apoptosis (El Kebir et al., 2012; Herrera et al., 2015; Artiach et al., 2018), and also attenuates APC functions targeting dendritic cell migration and reducing IL-12 production via ChemR23 (Arita et al., 2005a). Furthermore, RvE1 can restore inflammation induced mitochondrial dysfunction and reduce polymorphonuclear leukocyte infiltration in BLT1 dependent manner (Arita et al., 2007; Mayer et al., 2019), along with ChemR23-mediated counter regulatory actions to mediate the resolution of inflammation. It is also evident that RvE1 suppresses inflammatory cytokine release, facilitating the healing process, and inhibits macrophage migration by activating ChemR23 in a ligation model of acute MI (Liu et al., 2018). Moreover, supplementation of *Apoe^−/−^* mice with polyunsaturated fats as potentially beneficial intervention was supposed to enhanced interaction of RvE1 with ChemR23 in atherosclerosis prone mice and reduced their lesion size (Laguna-Fernandez et al., 2018). However, one study proposed that RvE1 and chemerin compete for the same recognition site on ChemR23, and RvE1 binding is blocked at the presence of the chemerin peptide (Arita et al., 2005a). Whether above summarized results hold true when RvE1 is not actively administered or otherwise supplemented, most likely exceeding competition for the recognition site on ChemR23 by chemerin, has yet to be fully elucidated.

Taken together, all these findings open a potential new avenue for the modulation of the magnitude of the local inflammatory responses also in chronic inflammatory disease such as atherosclerosis by fine tuning receptor specific responses in a cell and tissue specific manner.

### Concluding Remarks

The chemerin/ChemR23 axis is a complex network involved in the regulation of immune responses contributing to both the onset and the termination of inflammation. However, several studies show that the various chemerin isoforms may exert different actions downstream of ChemR23. Since chemerin has multiple and different actions, the possibility to selectively modulate its activity can become an attractive target for drug development. Thus, the use of substances boosting its anti-inflammatory properties could be a promising target in exploiting new strategies to treat atherosclerosis (please refer to Table 1 for a summary of important studies and their key findings and to Table 2 for an overview of ligand types involved).

## Calcium-Sensing Receptor

The CaSR belongs to the metabotropic glutamate receptor GPCR subfamily and is most abundantly expressed in the parathyroid gland and kidney (Brown et al., 1993; Aida et al., 1995; Riccardi et al., 1995; Bockaert and Pin, 1999). Here it senses changes in extracellular Ca^2+^ concentrations and couples this to intracellular signaling pathways that modify parathyroid hormone (PTH) secretion and renal calcium reabsorption in order to maintain the Ca^2+^ homeostasis (Diez-Fraile et al., 2013).

### Ligands and Signaling

Although Ca^2+^ is the main agonist for this receptor, CaSR responds to several other cations (e.g., Mg^2+^, Gd^2+^, Sr^2+^, La^2+^, and Ba^2+^) and a variety of other ligands (McLarnon and Riccardi, 2002). Interestingly, Mg^2+^ has the potency to augment CaSR signaling responses in the presence of Ca^2+^ and thus is a positive allosteric modulator, meaning that serum Mg^2+^ will affect Ca^2+^-CaSR signaling in clinical conditions (Ruat et al., 1996). Moreover, Mg^2+^ has been shown to also stimulate CaSR mRNA expression and protein levels whilst CaSR activation decreases Mg^2+^ levels (Ikari et al., 2001), indicating a negative feedback loop between Mg^2+^ and CaSR. Other, non-cation agonists of the receptor include polyamides, such as spermine and spermidine, and various amino acids (Quinn et al., 1997; Conigrave et al., 2000).

Upon ligand binding, CaSR activates several intracellular signal transduction pathways, mainly through Gαi, Gαq, and Gα12/13 G-protein subtypes. CaSR influences several effectors, such as PLC, adenylate cyclase (AC), cytosolic phospholipase A2 (cPLA2), phosphatidylinositol 4-kinase (PI4K), phospholipase D (PLD), and ERK (Kifor et al., 1997; Chang et al., 1998; Arthur et al., 2000; Huang et al., 2002; Huang et al., 2004).

### CaSR in Inflammatory Diseases

Abnormal CaSR activity or expression contributes to the development of CVDs. Following the discovery that CaSR is expressed in the heart tissue of rats, Guo et al. (2012) investigated the relationship between CaSR and MI in atherosclerosis by inducing MI in atherosclerotic rats and non-atherosclerotic controls. Here the authors showed that CaSR expression was significantly increased in the atherosclerotic MI group compared to the MI controls, suggesting that CaSR plays an important role in MI caused by atherosclerosis (Guo et al., 2012). Besides its suspected role in atherosclerosis and MI, CaSR is also important in numerous other inflammatory diseases. For example, a population-wide study and Felderbauer et al. (2006) linked several CaSR polymorphisms and mutations to chronic pancreatitis (CP) and idiopathic CP (Muddana et al., 2008). Furthermore, a study focusing on asthma showed that asthmatic patients and allergen-sensitized mice have higher expression levels of CaSR (Yarova et al., 2015), linking abnormal CaSR expression to yet another inflammatory disease. Moreover, Cheng et al. (2014) reported a diminished intestinal barrier function and a more inflammatory immune response in intestinal epithelial-specific CaSR knockout mice, all of which increased their susceptibility to chemically induced colitis. Together, these studies clearly show a very broad involvement of CaSR in inflammatory processes.

### Monocyte-Specific CaSR

CaSR is expressed on various inflammatory cells, such as monocytes and macrophages. Expression of CaSR on monocytes has been implicated in chemotaxis, a key process in inflammatory diseases. For example, one study indicated an interrelationship between CCR2 and CaSR and also showed that Ca^2+^ stimulates the chemotaxis of monocytes to CCL2 in a CaSR-dependent manner (Olszak et al., 2000). Paccou et al. (2013) followed up on this study by investigating CaSR expression on monocytes in response to several stimuli. Here, total CaSR expression increased in monocytes upon calcitriol, the biologically active form of vitamin D, stimulation whilst TNF decreased total CaSR expression in a dose-dependent manner (Paccou et al., 2013). Further connecting this receptor to inflammatory diseases, several studies report the activation of the NLRP3 inflammasome by CaSR in monocytes, where the review by Tang et al. (2018) provides an overview of all GPCRs involved in NLRP3 inflammasome activation and inhibition. NLRP3 inflammasome activation consequently leads to caspase-1 activation, which in turn cleaves pro IL-1β and pro IL-18 into their active forms mediating a pro-inflammatory response (Groslambert and Py, 2018). A study by Rossol et al. (2012) showed that monocytes sense changes in extracellular Ca^2+^ concentrations via CaSR signaling, which subsequently leads to the activation of the NLRP3 inflammasome (Rossol et al., 2012). However, another study showed that NLRP3 is also activated by decreased cAMP concentrations, which is in striking contrast to the study by Rossol et al. (2012) where no significant influence of cAMP levels on inflammasome activation could be detected (Lee et al., 2012). Also in a human setting, CaSR could already be linked to atherosclerosis development as Malecki et al. (2013) showed a 1.5-fold increased CaSR expression on peripheral blood monocytes of patients with peripheral artery disease (PAD). Overall, CaSR expression on monocytes seems to enhance pro-inflammatory responses via stimulation of chemotaxis and inflammasome activation.

### Macrophage-Specific CaSR

Focusing more on macrophage-specific CaSR, stimulation of CaSR promotes the release of pro-inflammatory mediators, such as IL-1β and TNF-α, by monocyte derived macrophages (Xi et al., 2010). In line with this, Canton et al. (2016) reports that extracellular Ca^2+^ is sensed by CaSR which subsequently signals through PLC and PI3K to induce constitutive micropinocytosis. Interestingly, two studies also linked macrophage-specific CaSR to the activation of the NLRP3 inflammasome. One study investigated the causal role of CaSR in the activation of the NLRP3 inflammasome via proteolytic pathways. It showed that the receptor activates the NLRP3 inflammasome and proteolytic maturation of IL-1β in differentiated macrophages via a chaperone-assisted degradative pathway (Gutierrez-Lopez et al., 2018). Another study by Lee et al. (2012) focused on the role of extracellular cations in inflammasome activation. It showed that CaSR activates the NLRP3 inflammasome in bone marrow-derived macrophages via increased intracellular Ca^2+^ and decreased cAMP (Lee et al., 2012). Together, CaSR expressed on macrophages also promotes pro-inflammatory responses via increased release of cytokines and inflammasome activation.

### Pharmacological Intervention

As CaSR is implicated in various diseases, it has become an interesting pharmacological target to investigate. The compounds which target the receptor can be divided into two categories: the calcimimetics and calcilytics. Calcimimetic compounds are positive allosteric modulators and include Cinacalcet, NPS R-467 and NPS R-568 (Nemeth et al., 1998, 2004). On the other hand, calcilytics such as NPS 2143, Ronacaleret and Calhex 231 are negative allosteric modulators (Nemeth et al., 2001; Petrel et al., 2003; Balan et al., 2009). Cinacalcet was the first allosteric GPCR modulating compound to be approved for the market (Brauner-Osborne et al., 2007), showing promising results in several case studies. One study reports durable and robust effects of Cinacalcet therapy in patients with neonatal severe hyperparathyroidism (NSHPT). Cinacalcet was offered as an experimental alternative drug in a case of NSHPT, where Cinacalcet was successful in rapidly normalizing the patient’s serum calcium levels, thereby improving muscle tone and the overall clinical condition (Gannon et al., 2014). Another study reports an acute increase of urinary calcium excretion in renal transplant recipients with secondary hyperparathyroidism after treatment with Cinacalcet, without showing adverse effects on glomerular filtration rate or renal graft calcium deposits (Courbebaisse et al., 2012). Calcilytics are mostly researched as a potential treatment of osteoporosis, but with limited success. Although clinically safe, no calcilytic to this day has been approved to be used to treat osteoporosis in humans (Kiefer et al., 2011). However, research has suggested the use of calcilytics in other diseases than osteoporosis, as for example Yarova et al. (2015) showed that calcilytics abrogate airway hyperresponsiveness and inflammation in allergic asthma.

### Concluding Remarks

Overall, CaSR plays an important role in various chronic inflammatory diseases, which is underlined by the many pro-inflammatory mechanisms induced by CaSR signaling in monocytes and macrophages. Calcimimetics and calcilytics show great therapeutic potential in other disease types, suggesting a potential of these drugs in the treatment of chronic inflammatory diseases as well. Important to keep in mind is that certain mutations and polymorphisms of CaSR affect the binding affinity of its allosteric modulators (Leach et al., 2013), making various patients less sensitive to pharmacological intervention. The development of compounds which can overcome these obstacles should be a focus point in future pharmacological research (please refer to Table 1 for a summary of important studies and their key findings and to Table 2 for an overview of ligand types involved).

## Closing Remarks

Research in recent decades has improved our understanding of the complex mechanisms of inflammatory processes within atherosclerosis. In addition, the CANTOS trial has clearly shown that the reduction of inflammatory processes has a positive effect on the outcome of CVD. The latter is particularly true for patients with pre-existing inflammatory conditions. In the context of these inflammatory processes, the interaction and activation of immune cells plays an important role. GPCRs are a group of receptors that play a central role in controlling these immune responses, but they are ubiquitously expressed and convey both pro- and anti-inflammatory signals. In addition, many of these receptors detect different ligands, which in turn deliver diverse immune responses depending on context (acute versus chronic), tissue and cell type involved. For example, the expression of CXCR4 seems atheroprotective by retention of neutrophils in the bone marrow and by maintaining arterial endothelial integrity while simultaneously endothelial-derived CXCL12 (*bona fide* ligand of CXCR4) appears pro-atherogenic. Data on FPR2 are similarly contradictory; here *in vivo* studies in mice show both more and less plaque progression in case of deletion of FPR2. In the context of an Annexin A1 supplementation, however, protective effects of the interaction of Annexin A1 and FPR2 on myeloid cells have been described. These examples, in turn, underline the significance of a specific ligand and cell type as part of a particular immune response. It is therefore crucial, in addition to the further characterization of receptor–ligand interactions and their consequences within chronic inflammation, to not draw generalized conclusions, but focus on individual conditions. With regard to therapeutic intervention aimed at GPCR-mediated immune responses, it is therefore also crucial to improve cell-specific drug delivery approaches and to identify other potentially impacting factors such as variation of genetic or epigenetic factors, which may influence therapeutic outcomes. Eventually, improvement of CVD therapy with respect to effective but safe therapeutics does clearly point in the direction of a cell-specific treatment tailored to the individual patient. Further elucidation and understanding of the concept of biased ligands, resulting in different signaling and thus effects of the binding of distinct ligands to the same receptor, could further improve the development of tailored treatment.

## Author Contributions

EvdV, LP, MM, SG, YY, and YD drafted the manuscript and made critical revisions. CW made critical revisions.

## Conflict of Interest Statement

The reviewer RK declared a shared affiliation, with no collaboration, with the authors EvdV, CW to the handling Editor at the time of review. The remaining authors declare that the research was conducted in the absence of any commercial or financial relationships that could be construed as a potential conflict of interest. The reviewer SL declared a past co-authorship with several of the authors to the handling Editor.
